# Optically Transparent Frequency Selective Surfaces with Wideband Capability for IoT Applications: A Polarization-Independent Double-Layer Design

**DOI:** 10.3390/s24144724

**Published:** 2024-07-21

**Authors:** Omer Faruk Gunaydin, Sultan Can

**Affiliations:** 1 Robert Bosch GmbH, Bursa 16140, Turkey; 2 Department of Electrical and Electronics Engineering, Ankara University, Ankara 06830, Turkey

**Keywords:** double-layer, frequency selective surfaces, optically transparent, polarization insensitive, smart cities, wide-band

## Abstract

This study proposes wide-band frequency selective surfaces (FSS) with polarization-independent characteristics that are tailored for IoT applications. The design consists of two different layers with band-stop characteristics that target key frequency bands in sub-6 GHz: 3.7 GHz (n77) and 4.5 GHz (n79), offering a 1.39 GHz bandwidth spanning from 3.61 GHz to 5.0 GHz. This study also presents a double-layer structure with a WB property with a fractional bandwidth of 32%. Simulations have been conducted to observe variations in insertion loss across incident and polarization angles ranging from 0 to 60 degrees for both TE and TM modes in the suggested FSS structures. These simulations demonstrate the design’s polarization independence. Transparent polyvinyl chloride with a dielectric constant of 2.77 and a thickness of 1.48 mm has been utilized as the substrate material. The optical transmittance is calculated to be 96.7% for Layer 1, 95.7% for Layer 2, and 92.4% for the double-layer structure, and these calculated optical transmittance values were found to be higher compared to the studies in the literature. The proposed design is well-suited for sub-6 GHz IoT applications due to their high transparency, cost-effectiveness, robust high-performance capabilities in suppression, and polarization-independent features. The results of 3D full-wave simulations were compared with measurement and the equivalent circuit model outcomes, and a good agreement between the results was observed.

## 1. Introduction

Rapid development in a smart city model, supported by an array of cutting-edge technologies and methodologies, bolsters models with IoT, delivering smart services that enhance performance and operations across healthcare, transportation, energy, education, and other key domains [1,2]. Smart city technology requires optimizing resource utilization, enhancing city infrastructure efficiency, and elevating the overall quality of urban life for residents. Communication is one of the most critical factors in achieving quality [3]. Originally envisioned as the primary frontier for the IoT and occasionally hailed as the Internet of Everything (IoE), fifth-generation (5G) wireless communication faced certain limitations, prompting researchers to seek a technological leap forward. This led to the pursuit of enhanced network capacity and reliability, an aim targeted by sub-6 GHz and sixth-generation (6G) wireless communication technologies [4], delivering expansive coverage over wide areas and supporting applications that demand extensive coverage contribute to quality as well. Within the electromagnetic (EM) spectrum, the sub-6 GHz band provides superior coverage and penetration compared to the higher-frequency bands [4,5].

Communication equipment and systems encounter diverse forms of EM influences throughout their entire operational lifespan. Non-compliance with electromagnetic compatibility (EMC) and electromagnetic interference (EMI) conditions results in equipment damage and incorrect operation such as failures or unnecessary alarms in protection devices. The usage density of those 5G and sub-6G devices, such as multiple-input–multiple-output (MIMO) and reconfigurable antennae, in the smart city has made EMI and EMC significant concerns for people [6]. It is not just about eliminating, suppressing, or filtering electromagnetic (EM) wave interactions between devices but also about addressing interactions between humans and devices. Frequency-selective surface (FSS) structures, as spatial filters, possess multi-function properties, including enhanced oblique stability, polarization insensitivity, and wide/ultra-wide bands [7,8,9].

Targeting multi-functional designs for IoT applications, this study presents two individual single- and double-layer designs, ensuring an optimal balance between size, cost, and functionality, such as enhanced bandwidth and transparency. The proposed designs boast exceptionally high optical transparency, remain polarization-independent, and offer cost-effective solutions. This paper demonstrates the equivalent circuit model of the proposed single-layer designs. The resonance frequency, inductance, and capacitance values are calculated according to the geometry of the design and the dielectric material in Section 2. The calculated resonance frequency is compared with the simulation and measurement results. In Section 3, parametric analyses are performed for single-layer and double-layer designs. Within the scope of parameter analysis, the effects of the dielectric material used, circle diameter, patch thickness, and incident and polarization angle (TE and TM) change on the proposed design were examined.

## 2. FSS Design Topology and Equivalent Circuit Model

FSSs integrated into everyday surfaces stand as pivotal components for IoT applications. They enable the intelligent manipulation and filtering of radio waves, facilitating independent interaction between objects and their surrounding environments [10,11]. FSS, also known as spatial filters, serve to alter incident electromagnetic waves, offering dispersive characteristics in their transmission and reflection [12,13]. The emphasis on spatial filtering highlights the significance of FSSs in research due to their diverse potential applications, including absorbers [14], dichroic sub-reflectors, radomes [15], RFIDs, shielding [16,17], and EMI protection [18]. These surfaces exhibit various filtering characteristics such as band-pass, band-stop, low-pass, and high-pass [12]. Among these, low-pass and high-pass FSS filters are prevalent, with the former allowing for lower frequency ranges while obstructing higher frequencies, and the latter operating as a low-pass filter [19]. Stop-band FSS is utilized to block unwanted frequencies, while pass-band FSS filters permit a specific frequency range while rejecting others. Resonance in FSSs is achieved through periodic arrays of metal patches and/or slots etched onto a dielectric material.

**Figure 1:**
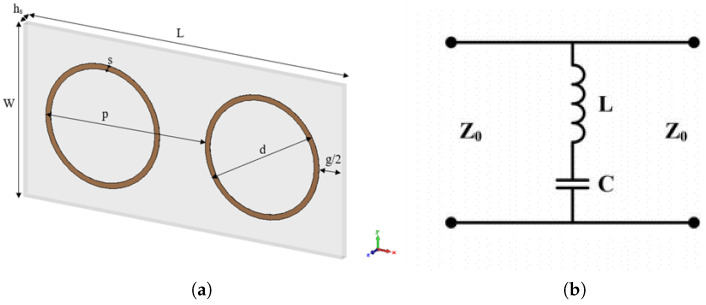
Proposed designs: (**a**) single-layer architecture; (**b**) equivalent circuit model.

The topology studied in this paper is depicted in Figure 1a from the top view of the unit cell design. For fabrication and measurement simplification, the proposed structure is considered to fit in a waveguide WR229 and the fundamental parameters for individual designs are presented in the following sections. Transparent polyvinyl chloride (PVC) has been used as the substrate material at the bottom, with a dielectric constant ($\epsilon_{r}$) of 2.77, and it is optically transparent in the visible region. The conductor segment of the proposed design constitutes a circular loop resonator, commonly recognized for its diameter, denoted as “d”. In the conductive part, copper conductor stripes with a thickness of 35-μm have been utilized. Dimensional parameters of FSS are validated during the design phase through calculations of equivalent circuit models based on the design dimensions.

Circuit models are beneficial for enhancing the comprehension of the frequency response of FSS [7]. The equivalent circuit model (ECM) provides a swift and straightforward approach for analyzing FSS, serving as a viable substitute for comprehensive wave simulations. This model operates on a transmission line comparison, representing the FSS through equivalent lumped components of inductance (*L*) and capacitance (*C*) [20,21]. Capacitance arises from the accumulation of electric charge in the gaps between adjacent loops of the conducting material, while inductance occurs as a result of current flowing around these adjacent loops [8]. The calculations for the *L* and *C* values stem from Markuvitz’s development of a quasi-static ECM approximation specifically tailored to the conducting strips [22]. Figure 1b illustrates the ECM of these FSSs, featuring an *LC* circuit where conducting elements and inter-element spacing symbolize *L* and *C*, respectively. As a result, the resonant frequency is determined by [23] as follows:

(1) $$f=\frac{1}{2\pi \sqrt{LC}},$$

Thefractional bandwidth (*FBW*) is indicated by the variance between the upper and lower frequency responses, both measured by considering the −10 dB threshold, presented as follows:

(2) $$FBW=\frac{f_{u}-f_{l}}{f_{0}}\times 100\%,$$

where $f_{u}$ refers to the upper frequency, $f_{l}$ refers to the lower frequency (considering the −10 dB threshold), and $f_{0}$ refers to the center frequency. Utilizing the ECM, the equivalent inductance and capacitance are derived from specific physical parameters characterizing an FSS. These parameters, including periodicity (*p*), loop length (*d*), strip width (*s*), angle of incidence (θ), and inter-loop gap *(g),* are depicted in Figure 1a. The typical approach for calculating both *L* and *C* values within the equivalent circuit (EC) involves Equations (3) and (4), given by [21,22].

(3) $$\frac{X_{L}}{Z_{0}}=\omega L=\frac{d\cos\theta F(p,2s,\lambda ,\theta )}{p},$$

(4) $$\frac{B_{C}}{Y_{0}}=\omega C=4\frac{d}{p}\sec\theta F\left(p,g,\lambda ,\phi \right)\varepsilon_{\mathrm{eff}},$$

where

(5) $$F\left(p,w,\lambda ,\theta \right)=\frac{p}{\lambda }\left[\ln\left(\csc\left(\frac{\pi w}{2p}\right)\right)+G\left(p,w,\lambda ,\theta \right)\right],$$

(6) $$G\left(p,w,\lambda ,\theta \right)=\frac{1}{2}\times \frac{{\left(1-\beta^{2}\right)}^{2}\left[\left(1-\frac{\beta^{2}}{4}\right)\left(A_{+}+A_{-}\right)+4\beta^{2}A_{+}A_{-}\right]}{\left(1-\frac{\beta^{2}}{4}\right)+\beta^{2}\left(1+\frac{\beta^{2}}{2}-\frac{\beta^{4}}{8}\right)\left(A_{+}+A_{-}\right)+2\beta^{6}A_{+}A_{-}},$$

with

(7) $$A_{\pm }=\frac{1}{\sqrt{\left[1\pm \left(\frac{2p\sin\theta }{\lambda }-{\left(\frac{p\cos\theta }{\lambda }\right)}^{2}\right]\right)}}-1,$$

(8) $$\beta =\sin\left(\frac{\pi w}{2p}\right).$$

The equation relates to angles of incidence (θ and ϕ), wavelength (λ), and a correction term denoted as G in [21,22,23]. Munk [19] introduced $\epsilon_{eff}$ with a value of 0.5 ($\epsilon_{r}$ + 1), applied in the literature for square-loop FSS. This parameter acts as an effective means by which to modify the dielectric permittivity. However, after conducting numerous simulations, it was observed that the accuracy of including or excluding this adjustment factor applies specifically to scenarios where the dielectric substrate is either significantly thick (>λ/5) or extremely thin (<λ/100), respectively.

Neglecting correction factors, albeit introducing a slight bias in the outcome, allows for the rewriting of Equations (3) and (4) as follows:

(9) $$\frac{\omega_{r}L}{Z_{0}}=\frac{d}{p}\cos\theta \times \frac{p}{\lambda }\ln\left[\csc\left(\frac{\pi w}{2p}\right)\right],$$

(10) $$\frac{\omega_{r}C}{Y_{0}}=4\frac{d}{p}\sec\theta \times \frac{p}{\lambda }\ln\left[\csc\left(\frac{\pi g}{2p}\right)\right]\times \varepsilon_{\mathrm{eff}}$$

Considering the topology that is presented in Figure 1a, Table 1 provides the parameter values for each layer. The substrate has a width of W with a value of 29.083 mm, a length, denoted as L, with a value of 58.166 mm, and a thickness, denoted as $h_{s}$, with a value of 1.48 mm. The double-layer structure is the combination of these two layers with an 8 mm gap separation, with one layer in the front and the other in the backside of this cascade structure.

Within this section, the calculation of lumped elements (L and C) for individual layers is performed using the parameter values outlined in Table 1. The resonance frequency was determined after calculating the L and C values using the ECM for the layers. For Layer 1, the parameters were p = 29.083, d = 20.8, g = 8.283, and s = 0.9 mm. These values led to a calculated resonance frequency of 4.53 GHz, with corresponding C and L values of 0.048 pF and 25.74 nH, respectively. Layer 2 exhibited a calculated resonance frequency of 3.77 GHz, with an L value computed at 28.60 nH and a C value at 0.059 pF. Through simulations using the CST full-wave simulator, the observed resonance frequency for Layer 1 was 4.51 GHz. Meanwhile, the equivalent circuit calculations yielded a resonance frequency of 4.53 GHz. In the simulation result, the resonant frequency for Layer 2 was determined to be 3.69 GHz, while the ECM indicated a resonant frequency of 3.77 GHz. It can be observed that the resonant frequency calculated using the ECM closely matches the simulation results for both layers.

## 3. Analysis, and Evaluation of the Final Design

In this section, the details of the unit cell designs, full-wave simulations, and analyses are conducted and presented. Studies were conducted for the single-layer design, and to enhance the bandwidth of the final design, they were extended to the double-layer design.

### 3.1. Single-Layer Designs

**Figure 2:**
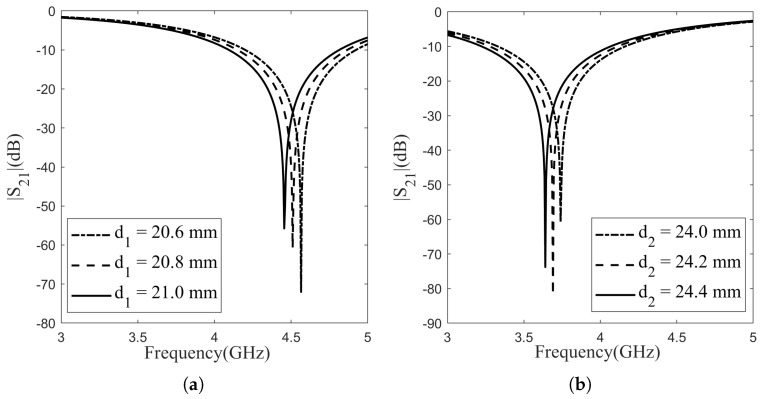
Effect of diameter changes to frequency characteristic: (**a**) Layer-1; (**b**) Layer-2.

**Figure 3:**
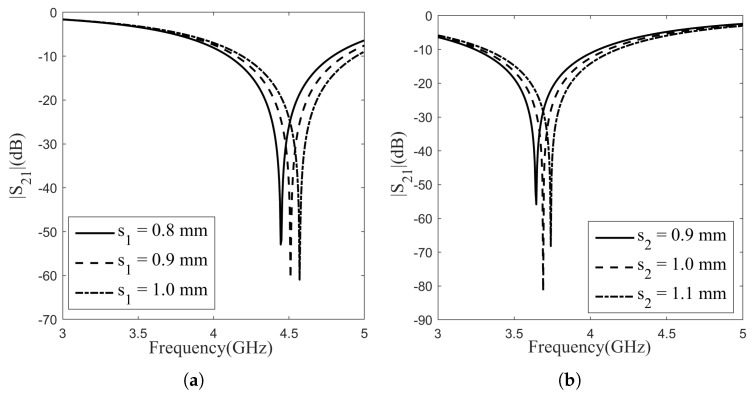
Effect of patch width to frequency characteristic: (**a**) Layer-1; (**b**) Layer-2.

The aforementioned topology comprises two distinct layers, each tailored for specific frequency points. Both designs are aligned and fit into the WR229 waveguide, and the substrate material employed is PVC, as mentioned above. The FSS’s frequency response is primarily dictated by the structural shape and its dimensional attributes. Among these, pivotal dimension parameters influencing the efficacy of FSS filters include the loop length “d” (diameter for circular loops), the loop width “s”, the unit cell periodicity “p”, and the gap between the loops “g”. These parameters play a crucial role in determining the FSS’s performance characteristics. The impact of varying the diameter and loop width of the circular FSS element on the frequency characteristic was investigated for each layer, as depicted in Figure 2 and Figure 3. Across layers, full-wave simulations were conducted by varying the diameter of the circle with a step size of 0.2 mm, and the simulation results for Layer 1 are shown in Figure 2a, while for Layer 2, they are depicted in Figure 2b. These adjustments were analyzed to assess their impact on the resonance frequency and bandwidth. In Layer 1, enlarging the circle diameter led to an approximate 0.05 GHz decrease in resonance frequency, while the bandwidth remained consistent at 0.7 GHz across all diameter increments. Layer 2 demonstrated resonance frequency behavior akin to Layer 1, also decreasing by 0.05 GHz for every 2 mm diameter augmentation. Likewise, the bandwidth maintained a uniform value of 0.8 GHz across the three cases. Full-wave simulations were carried out across layers, altering the patch width of the circle in increments of 0.1 mm. The simulation outcomes for Layer 1 are displayed in Figure 3a, while those for Layer 2 are presented in Figure 3b. In Layer 1, widening the patch led to an approximate 0.07 GHz increase in resonance frequency, while the bandwidth remained consistent at 0.7 GHz for all increments in diameter. Layer 2 displayed resonance frequency behavior akin to Layer 1 and increased by 0.05 GHz for every 0.1 mm diameter increment. Similarly, the bandwidth maintained a uniform value of 0.8 GHz across all three scenarios. Simulations indicate that increasing the diameter of the circle or the patch width does not have any effect on bandwidth but as the circle diameter increased, the resonant frequency decreased, whereas with an increase in the patch width, the resonant frequency increased.

**Figure 4:**
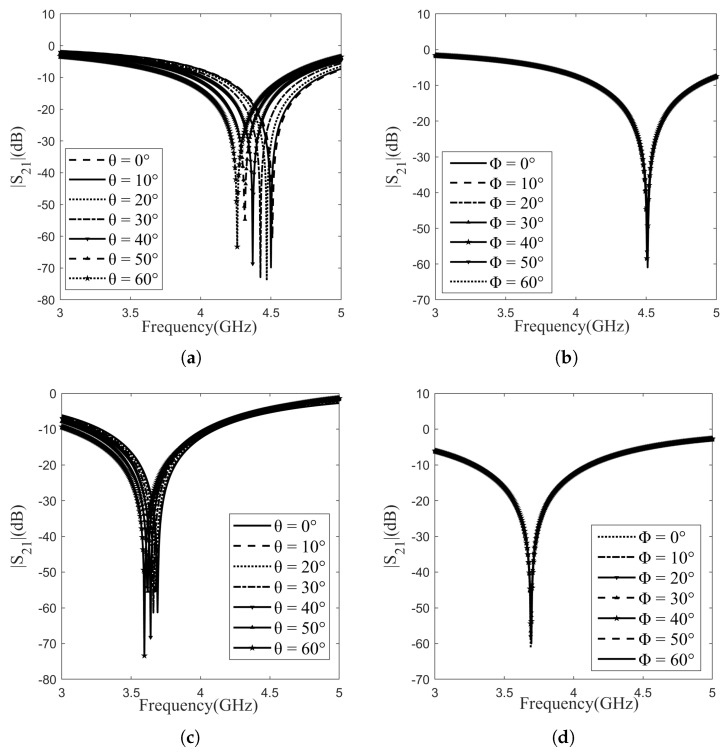
Polarization TE and TM mode: (**a**) Layer-1 (θ); (**b**) Layer-1 (Φ); (**c**) Layer-2 (θ); (**d**) Layer-2 (Φ).

The impact or polarization angle (Φ) independency and angle of incidence (θ) on the performance was also evaluated. Assuming a linearly polarized wave with the E-field in the direction of the y-axis that strikes the periodic cell at normal incidence, i.e., an incident E-field, the geometrical parameters of the cells can be adjusted to control the operating frequencies of the surfaces. The same applies when the incident E-field is oriented in the direction of the x-axis. By proposing a symmetric design, identical responses for both TE and TM modes are obtained since asymmetrical patterns have a disruptive effect on the performance of the FSSs. Figure 4 illustrates the changes in insertion loss observed in the proposed FSSs across angles of incidence and polarization ranging from 0 to 60 degrees for both TE and TM modes, respectively. As depicted in Figure 4b,d, layers exhibited the same response for the TM and TE mode, remaining unaffected by the angle of polarization ranging from 0 to 60 degrees in increments of 10 degrees.

Figure 4a,c display the variations in insertion loss observed in the suggested FSSs across incident angles, spanning from 0 to 60 degrees for the TE and TM modes. The response of Layer 2 for the TE mode showcases a shift of approximately 0.015 GHz in resonance frequency for every 10-degree increment. However, in Layer 1, this frequency shift was determined to be around 0.05 GHz for every 10-degree increase. Across all layers, it was observed that the focused frequency and bandwidth (−10 dB) remained unchanged for both TE and TM modes. In conclusion, simulations revealed that variations in angle had no discernible impact on magnitude, bandwidth, or resonance frequency for both TE and TM modes. This demonstrates that the proposed designs are independent of polarization. The proposed FSS successfully maintains nearly identical responses for both TE and TM polarization, eliminating the need for any structural modifications when used for either polarization. The FSSs demonstrated stable behavior throughout this range of incident angles and polarization angles.

**Figure 5:**
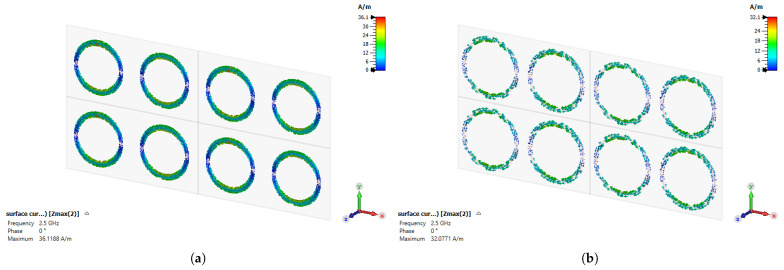
Surface current density: (**a**) Layer-1; (**b**) Layer-2.

The surface current distribution in frequency-selective surfaces refers to the distribution of electric current on the surface. This current distribution determines the electromagnetic properties of the surface structure and affects the characteristics of electromagnetic waves that are either emitted or absorbed [24]. Surface current is a fundamental factor determining the performance of frequency-selective surfaces because it influences the behavior, permeability, and resonance properties of the surface [25]. Therefore, surface current, by determining the electromagnetic behavior of the surface, is a critical measure that defines the operating principles and performance of frequency-selective surfaces. In Figure 5a, surface current distributions are illustrated for Layer 1 when excited by a normal incident wave. In Figure 5b, the surface current distributions for Layer 2 under normal wave excitation are depicted. When observing layers, it is evident that the surface current density is higher at the top and bottom sections of the circle and notably lower at the sides because of the coupling among those edges.

The PVC substrate exploited in the design is 100% transparent. Yet, exploited conducting copper layers limit the transparency level of the proposed topology. The transparency of the structures is calculated by subtracting the conducting area from the dielectric area. The transparency percentage for this limitation is computed using the following formula: ((total physical area − the area occupied by the conductor)/total physical area) × 100. Layer 1 exhibits an optical transparency of 96.7%, while Layer 2 demonstrates 95.7%. Comparative analysis with the existing literature reveals that the proposed design showcases notably high optical transparency values. The conductive part utilized copper with a thickness of 35 µm.

### 3.2. Double-Layer Design

**Figure 6:**
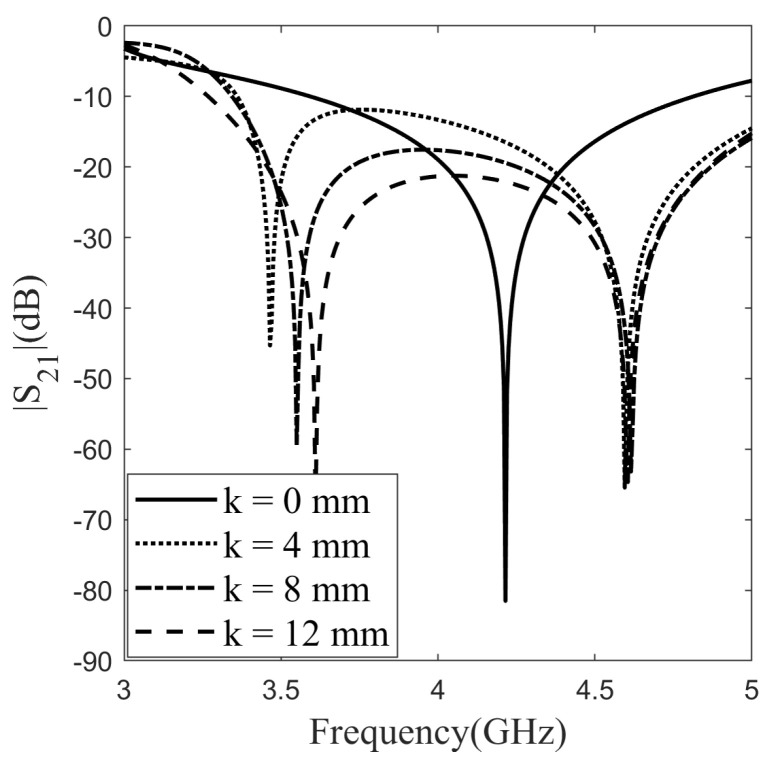
Effect of inter-layer distance to frequency characteristic.

Each layer detailed in the single-layer section has been combined to create a double-layer design for enhancing the bandwidth. The purpose of forming a double-layer design is to achieve a wideband and optically transparent FSS. Optical transparency calculated for each layer in previous sections has been computed for different layer combinations in this section. The optical transparency obtained for the Layer 1–Layer 2 combination is 92.4%. Regarding the results, it is evident that high optical transparency has been achieved even in the double-layer structure. For a mechanically adjustable version of the structure, the air gap layer is considered, and the air gap thickness, referred to as the distance between the layers, has been denoted as “k”, and simulation studies have been conducted for different values of “k” and various combinations of layers, as illustrated in Figure 6. Simulations were conducted with step size 4 mm between 0 and 12 for the “k” value, examining its effect on bandwidth and isolation. Upon detailed inspection in Figure 6, for instance, when “k” equals 0 mm, meaning that Layer 1 and Layer 2 are adjacent, the bandwidth measures 1.20 GHz. When “k” equals 4 mm, the bandwidth extends to 1.65 GHz, maintaining the same measurement for “k” values of 4 mm and 8 mm, while “k” at 12 mm shows a bandwidth of 1.69 GHz.

Comparing bandwidths between “k” values of 0 mm and 4 mm reveals an approximate difference of 0.45 GHz. For other “k” values, significant increases in bandwidth are not observed, but an increase in “k” correlates with improved isolation in the calculated frequency range (below −10 dB). For instance, with “k” at 4 mm, the minimum isolation value is −11.96 dB; at 8 mm, it is −17.65 dB; and at 12 mm, it is −21.32 dB. Based on these simulation results, a “k” value of 8 mm has been determined as the most suitable choice; however, the adjustment of this air gap is challenging because of physical stability. The rationale behind this decision is its provision of a good bandwidth, favorable isolation values, and a reasonable distance between layers for the intended applications.

## 4. Fabrication and Experimental Validation of Proposed FSSs

**Figure 7:**
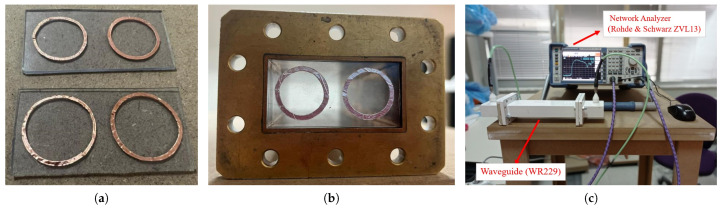
(**a**) Prototype of each layer; (**b**) single-layer fitted in the waveguide WR229; (**c**) measurement set-up.

To confirm the effectiveness of the optically transparent double-layer wideband design, Layer 1 and Layer 2 undergo fabrication through a process involving both printing and shearing techniques one by one. For a cost-efficient realization, conductive patterns are initially created using conductive copper tape, which is printed using an HP LaserJet P1005 printer. Subsequently, the conductors are precisely cut and affixed to predetermined positions on optically transparent PVC material, ensuring adherence to periodicity requirements. Layer 1 and Layer 2, manufactured manually for unit cells, are depicted in Figure 7a, while their representation in the waveguide is shown in Figure 7b. Measurements for each topology are carried out in the WR-229 waveguide with the Rohde and Schwarz ZVL13 vector network analyzer, as illustrated in Figure 7c. The samples are positioned in the middle of the waveguide vertically, supported by foams.

**Figure 8:**
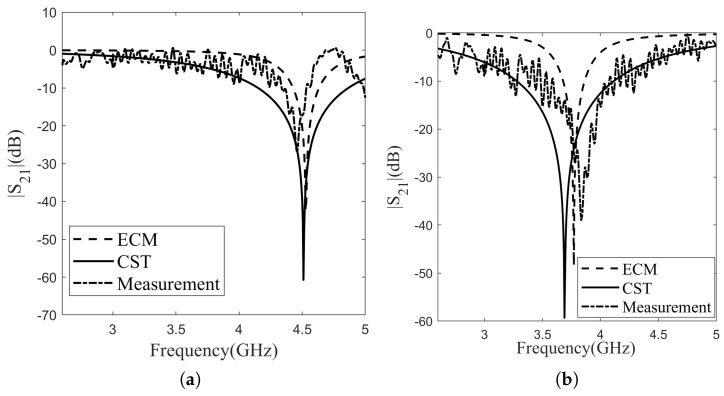
Comparison of measured results with the ECM and CST: (**a**) Layer-1; (**b**) Layer-2.

**Figure 9:**
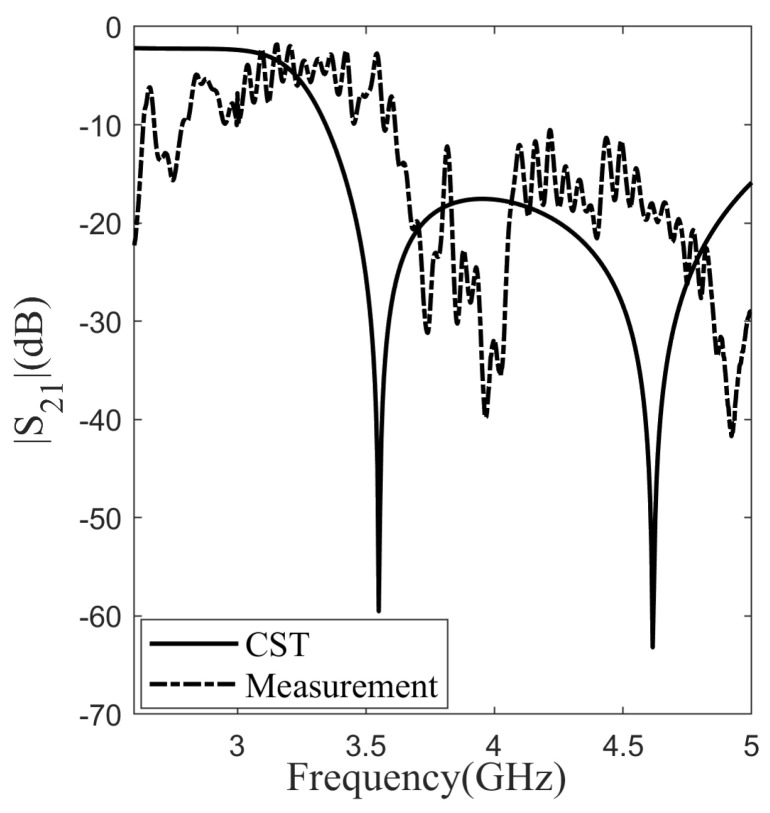
Comparison of measured results of double-layer structure with CST.

Measuring the transmission characteristics of the individual layers and double-layer is conducted using the WR-229 waveguide and the Rohde and Schwarz ZVL13 vector network analyzer. The measured transmission characteristics were compared with both simulation studies and the ECM. The measured magnitudes of the transmission coefficients are illustrated in Figure 8, accompanied by the outcomes of full-wave simulations and the ECM results (refer to Figure 8a for Layer 1 and Figure 8b for Layer 2). The measurement results, simulation outcomes, and ECM results for the resonance frequency have been presented in Table 2. The resonance frequency for Layer 1 is determined as 4.46 GHz in the measurement results, 4.53 GHz in the ECM results, and 4.51 GHz in the simulation results. Evaluating the error rates in comparison to the measurement results for the resonance frequency, the simulation results exhibit a deviation of 1.1%, while the ECM results show a deviation of 1.5%. In the measurement results, the resonance frequency for Layer 2 is identified as 3.83 GHz, 3.77 GHz in the ECM outcomes, and 3.69 GHz in the simulation results. Assessing the error rates relative to the measurement results for the resonance frequency, the simulation results demonstrate a deviation of 3.7%, whereas the ECM results indicate a deviation of 1.6%. For Layer 1, the upper- and lower-band operation frequencies are 4.34 GHz and 4.53 GHz, respectively. This indicates a bandwidth of 0.19 GHz for Layer 1. As for Layer 2, the upper- and lower-band operation frequencies are 3.6 GHz and 4.09 GHz, respectively, revealing a bandwidth of 0.59 GHz for Layer 2. When examining the results, it is observed that the measured resonance frequencies for both layers closely match the ECM and simulation outcomes. By employing Equation (2), the fractional bandwidth is calculated to be 4.1% for Layer 1 and 20.6% for Layer 2. Following the completion of measurements for the layers, two layers were placed inside the waveguide with a separation of 8 mm between them, and the transmission characteristics of the double layer were then measured. The measurement results and simulation outcomes for the double-layer design are presented in Figure 9. In the measurement results, the operating frequencies for the upper and lower bands are observed to be 3.61 GHz and 5.0 GHz, respectively, with a bandwidth of 1.39 GHz. Conversely, in the simulations, the upper and lower band frequencies are obtained as 3.39 GHz and 5.0 GHz, respectively, with a bandwidth of 1.61 GHz. A deviation of 0.22 GHz in the bandwidth was observed between the simulation and measurements. Due to the alignment disadvantages of the waveguide simulation setup, discrepancies arose between the simulated and measured results. Fabrication errors and mismatches in material parameters also contribute to these discrepancies.

The performances of the stop-bands and pass-bands are evaluated together, and the efficiency is discussed in terms of the absorption concerning frequency. Absorption for the pass-band is defined as Absorption = $1\;-\;|S_{11}{|}^{2}\;-\;{\left|S_{21}\right|}^{2}$, and the efficiency is defined as (Efficiency = 1 − Absorption). However, it should be noted that this loss may exceed the values that are presented here due to the inhomogeneous nature of everyday object materials such as the PVC used in the proposed designs.

The loss tangent, which is a measure of how much energy is lost as electromagnetic waves pass through or interact with the FSS material, can significantly impact the efficiency and performance of the surface. Various loss tangent values for Layer 1 and Layer 2 were examined regarding their impact on efficiency and absorption at specified frequencies, and the results are presented in Table 3. The loss tangent was analyzed starting from 0.02, with a step size of 0.02 up to 0.10, and it is seen that as the loss tangent increases, the absorption increases and the efficiency decreases accordingly. At resonance frequencies, especially near sharp resonance peaks, there can be heightened absorption due to the increased interaction and absorption of the incident electromagnetic waves by the FSS elements. For instance, Layer 2’s measured resonance frequency is 3.83 GHz. Table 4 demonstrates that for each loss tangent value, the highest absorption for Layer 2 occurs at 4 GHz, which is the measurement point nearest to the resonance frequency. The latest studies in the literature were considered and compared with the proposed study in terms of performance regarding parameters such as transparency, bandwidth, unit cell size, isolation values, number of layers, and polarization dependency. The results are presented in Table 4. When compared to those studies, the suggested FSSs provide the highest optical transparency and bandwidth for each layer and double layer. Moreover, considering the combination of the covered area and isolation, they demonstrate satisfactory performance compared to the rest. Additionally, thanks to the custom design process, the proposed design will facilitate cost-effective solutions. All individual layers and the double-layer structure are independent of polarization in both the upper and lower operation frequency bands.

## 5. Conclusions

This paper illustrates two distinct structures that are candidates for IoT applications because they offer the seamless integration and multifunctional capabilities, with features like ease of production, low cost, and high performance, and each possesses the ability to exhibit transparency in the visible spectrum. A bandwidth-enhanced double-layer design has also been presented for the use of the two most-used frequency bands below 6 GHz, namely, 3.7 GHz (n77) and 4.5 GHz (n79), exhibiting band-stop characteristics. When compared with the literature, the optical transparency of the proposed structures is significantly high for both single-layer and double-layer configurations. Additionally, the substrate material used is flexible (paving the way for cost-effective curved systems) and independent of polarization, making it suitable and practical for various applications. The independence of the proposed structure from polarization allows it to exhibit a stable characteristic for different incident angles. Thus, the design does not need to be repositioned even if the angle of the incoming electromagnetic wave changes, providing polarization-independent performance in the region where the design is applied. All the proposed designs are evaluated via CST and ECM, fabricated, and compared with the measurement results. A good agreement has been observed with the measured results.

## Figures and Tables

**Table 1:** Design parameters of individual layers.

Layer	p (mm)	d (mm)	g (mm)	s (mm)
1	29.083	20.8	8.283	0.9
2	29.083	24.2	4.883	1.0

**Table 2:** Resonance frequency comparison between simulation, ECM, and measurement.

Layer No.	Measurement Tool	Resonance Frequency (GHz)	Error Compared to Measurement
1	Measurement	4.46	-
	ECM	4.53	1.5%
	CST	4.51	1.1%
2	Measurement	3.83	-
	ECM	3.77	1.6%
	CST	3.69	3.7%

**Table 3:** Efficiency and absorption percentages of the proposed structures.

Layer	Loss Tangent	f = 3 GHz		f = 4 GHz		f = 5 GHz	
		Efficiency %	Absorption %	Efficiency %	Absorption %	Efficiency %	Absorption %
Layer 1	0.02	98.48	1.52	96.87	3.13	95.14	4.86
	0.04	97.08	2.92	94.14	5.86	91.20	8.80
	0.06	95.71	4.29	92.56	8.44	87.63	12.37
	0.08	94.37	5.63	89.13	10.87	84.38	15.62
	0.10	93.08	6.92	86.84	13.16	81.42	18.58
Layer 2	0.02	97.75	2.25	96.60	3.40	97.32	2.68
	0.04	95.74	4.26	93.58	6.42	95.08	4.92
	0.06	93.81	6.19	90.84	9.16	92.97	7.03
	0.08	91.96	8.04	88.29	11.71	90.96	9.04
	0.10	90.19	9.81	85.89	14.11	89.06	10.94

**Table 4:** Comparison between the proposed optically transparent double-layer with the state-of-the-art alternatives.

Reference	Number of Layer	$f_{L}/f_{U}$ (GHz)	U.C. Size ($\lambda_{0}\times \lambda_{0}$)	Polarization Insensitive $f_{L}/f_{U}$	Iso. (dB) $f_{L}/f_{U}$	Bandwidth (GHz) (−10 dB)	Transparency (%)
[9] (Top. A)	1	3.30/4.43	0.43 × 0.43	Yes/No	20/40	0.10/0.95	93
[9] (Top. B)	1	3.52/4.46	0.43 × 0.43	Yes/Yes	23/40	0.19/0.85	93
[11]	1	0.90/1.90	0.39 × 0.39	Yes/No	14/12	0.54/0.43	81.60
[17]	1	2.50/5.10	0.85 × 0.85	Yes/Yes	30/20	1.05/0.36	65.48
[26]	3	3.38/4.66	0.45 × 0.90	Yes/No	30/40	1.28	52.50
[27]	2	2.45/5.82	0.58 × 0.58	No/Yes	20/30	0.35/0.20	5
[28]	1	2.45/5.50	0.36 × 0.36	Yes/Yes	30/50	1.03/1.08	86
**TS**(L1)	1	4.34/4.53	0.44 × 0.44	Yes/Yes	28	0.19	96.7
**TS** (L2)	1	3.6/4.09	0.40 × 0.40	Yes/Yes	39	0.59	95.7
**TS** (DL)	2	3.61/5	0.48 × 0.48	Yes/Yes	39	1.39	92.4

$f_{L}$: the frequency of operation for the lower band; $f_{U}$: the frequency of operation for the upper band; Iso.: isolation in operating bands; $\lambda_{0}$: the wavelength guided at the center frequency of the upper band; L1: Layer-1; L2: Layer-2; DL: Double−layer; this study: **TS**.

## Data Availability

The dataset is available on request from the authors.
